# General Treatments Promoting Independent Living in Parkinson’s Patients and Physical Therapy Approaches for Improving Gait—A Comprehensive Review

**DOI:** 10.3390/medicina60050711

**Published:** 2024-04-25

**Authors:** Dae-Hwan Lee, Bong-Sik Woo, Yong-Hwa Park, Jung-Ho Lee

**Affiliations:** 1 IM Rehabilitation Hospital, 2140, Cheongnam-ro, Seowon-gu, Cheongju-si 28702, Chungcheongbuk-do, Republic of Korea; dhlee8510@naver.com (D.-H.L.); wbongsky@daum.net (B.-S.W.); rmsid0245@naver.com (Y.-H.P.); 2 Department of Physical Therapy, University of Kyungdong, 815, Gyeonhwon-ro, Munmak-eup, Wonju-si 26495, Gangwon-do, Republic of Korea

**Keywords:** Parkinson disease, gait, rehabilitation, physical therapy, RAGT

## Abstract

This study delves into the multifaceted approaches to treating Parkinson’s disease (PD), a neurodegenerative disorder primarily affecting motor function but also manifesting in a variety of symptoms that vary greatly among individuals. The complexity of PD symptoms necessitates a comprehensive treatment strategy that integrates surgical interventions, pharmacotherapy, and physical therapy to tailor to the unique needs of each patient. Surgical options, such as deep brain stimulation (DBS), have been pivotal for patients not responding adequately to medication, offering significant symptom relief. Pharmacotherapy remains a cornerstone of PD management, utilizing drugs like levodopa, dopamine agonists, and others to manage symptoms and, in some cases, slow down disease progression. However, these treatments often lead to complications over time, such as motor fluctuations and dyskinesias, highlighting the need for precise dosage adjustments and sometimes combination therapies to optimize patient outcomes. Physical therapy plays a critical role in addressing the motor symptoms of PD, including bradykinesia, muscle rigidity, tremors, postural instability, and akinesia. PT techniques are tailored to improve mobility, balance, strength, and overall quality of life. Strategies such as gait and balance training, strengthening exercises, stretching, and functional training are employed to mitigate symptoms and enhance functional independence. Specialized approaches like proprioceptive neuromuscular facilitation (PNF), the Bobath concept, and the use of assistive devices are also integral to the rehabilitation process, aimed at improving patients’ ability to perform daily activities and reducing the risk of falls. Innovations in technology have introduced robotic-assisted gait training (RAGT) and other assistive devices, offering new possibilities for patient care. These tools provide targeted support and feedback, allowing for more intensive and personalized rehabilitation sessions. Despite these advancements, high costs and accessibility issues remain challenges that need addressing. The inclusion of exercise and activity beyond structured PT sessions is encouraged, with evidence suggesting that regular physical activity can have neuroprotective effects, potentially slowing disease progression. Activities such as treadmill walking, cycling, and aquatic exercises not only improve physical symptoms but also contribute to emotional well-being and social interactions. In conclusion, treating PD requires a holistic approach that combines medical, surgical, and therapeutic strategies. While there is no cure, the goal is to maximize patients’ functional abilities and quality of life through personalized treatment plans. This integrated approach, along with ongoing research and development of new therapies, offers hope for improving the management of PD and the lives of those affected by this challenging disease.

## 1. Introduction

Parkinson’s disease (PD) is a neurological disorder primarily affecting the motor system, characterized by a variety of symptoms that can vary in severity among individuals. Here, we delve into the five primary symptoms: bradykinesia, muscle rigidity, tremors, postural instability, and akinesia [1].

In PD, these five main symptoms significantly restrict daily activities, leading to reduced natural movement, stiff posture, slower walking speeds, and difficulties in initiating and stopping movement. Additionally, these symptoms can affect facial expressions and voice modulation [1,2,3].

PD presents with a complex array of symptoms, often accompanied by other issues such as sleep disturbances, depression, or cognitive decline. Management typically involves a comprehensive treatment plan tailored to each individual’s needs [4].

PD is related to dopamine released by substantia nigra compacta. Dopamine plays an important role in basal ganglia functions. During movement, the basal ganglia modify information from the cerebral cortex, the brainstem, and the cerebellum to enable precise and delicate movement. They are regulated through direct and indirect pathways. As a neurotransmitter, dopamine is an essential mediator for the activation of direct and indirect pathways. Dopamine has an activating effect on behavioral conversion and continues to play a role in reward motor learning. In individuals with PD, damage to the neurons responsible for dopamine production in the basal ganglia of the brain results in a reduction in dopamine levels [4,5,6,7]. A deficiency in dopamine, which is crucial for regulating movement, is associated with impaired motor function. One of the main pathological mechanisms of PD involves the abnormal accumulation of alpha-synuclein and trophic-related proteins. This leads to neuronal damage and a decrease in dopamine production. Additionally, situations where the cells responsible for promoting dopamine production are impaired, or the cells that enhance dopamine degradation are overly active, can also contribute to a decrease in dopamine levels [5,6,7,8].

The symptoms of PD can vary in severity and type from patient to patient. Some patients only have mild symptoms, while others may have severe motor and neurological dysfunction. This diversity plays an important role in personalizing and optimizing treatment and rehabilitation methods [9]. PD causes a variety of symptoms related to motor control. These symptoms significantly reduce the patient’s quality of life, and the importance of appropriate treatment and rehabilitation is highlighted. Accordingly, it is necessary to develop personalized treatment and rehabilitation methods that consider the individual characteristics and symptoms of patients [10].

It was not until the late 1960s that dopamine replacement therapy became feasible, and as the century turned, deep brain stimulation surgery emerged as a viable surgical treatment option. These medical and surgical therapeutic options dramatically improved comprehensive symptom control and significantly extended the average lifespan of individuals with PD, often by decades [5,11]. These interventions effectively prolonged the period during which PD patients could live with the condition while managing its disabilities. As there is no cure for PD, and the condition continues to progress with significant disabilities, the role of effective physical therapy and rehabilitation management has become pivotal [12].

While the precise etiology of PD remains elusive, pharmacotherapy plays a crucial role in symptom management and disease progression [13]. This paper aims to elucidate the pharmacological agents commonly employed in the treatment of PD. Medications currently used for PD include levodopa, dopamine agonists, monoamine oxidase B (MAO-B) inhibitors, Catechol-O-Methyltransferase (COMT) inhibitors, and anticholinergics [14,15]. These medications are adjusted based on the patient’s symptoms and response, often employing a combination of drugs to achieve optimal effects. Therefore, treatment should be individualized [13,14,15].

Gait and balance abilities are crucial indicators used to assess the extent of recovery in patients with neurological damage, serving as important metrics of independent living [16]. Patients with PD often exhibit impairments in their gait abilities, including difficulties in initiating and stopping gait, shuffling gait, reduced joint mobility at the hip and knee due to abnormal muscle tension, and a tendency to assume a stooped posture [17]. The compromised balance abilities in Parkinson’s patients result from reduced proprioception and tactile sensation as well as difficulties in integrating sensory and muscular feedback [18]. Their reduced sense of balance also increases the risk of falls and postural instability, which also contributes to an increased risk of falls [19,20]. Currently, physical therapy for Parkinson’s patients includes electrical treatments such as functional electrical stimulation and electrical muscle stimulation (EMS) and neurorehabilitation models such as proprioceptive neuromuscular facilitation (PNF) and Bobath therapy [21]. In addition, aerobic training, resistance training, treadmill training, balance and gait training, and hydrotherapy are provided. Physical therapists improve the symptoms of Parkinson’s patients and restore their activities of daily living functions [22].

The impaired walking function and balance deficits in Parkinson’s patients result from disruptions in muscle coordination and tone [23]. One example of a treatment approach for Parkinson’s patients is progressive resistance training (PRT), a training method that gradually increases resistance applied to the muscles to enhance strength [24]. This method effectively strengthens the muscles of Parkinson’s patients, increasing lower limb strength, and aiding in the recovery of walking function [25]. Systematic research on such training has demonstrated the effectiveness of PRT in increasing strength after Parkinson’s onset [26].

Physical activity and exercise contribute significantly to preventing aging, preserving against chronic conditions, and enhancing fitness [27]. Numerous studies have shown that the inclusion of physical activities can reduce the incidence of neurological disorders such as stroke and PD. Exercise training has been linked to increased survival rates and the prevention of PD onset [28]. Exercise can stimulate brain function and neurogenesis by boosting various factors. Previous research has indicated that dopamine synthesis is enhanced due to serum calcium transported to the brain post exercise [29]. Dopamine levels can be influenced by physiological changes and physical activity [5,29]. Exercise has been demonstrated to lead to an increased brain structure for learning and memory, promoting survival [30]. Exercise is generally employed to enhance motor abilities and improve adaptability based on clinical evidence in PD patients [1,17]. It stimulates dopamine synthesis in the remaining dopaminergic neurons and enhances neuroplasticity, leading to symptom reduction [31]. Aerobic exercise during a resting state enhances functional plasticity, while exercise training on tracks improves interregional connectivity through motor learning [32].

Comparing walking training conducted on regular ground to treadmill-based walking training, it is reported that treadmill-based training is more effective [33]. Consequently, the importance of treadmill-based walking training has increased. Recently, task-oriented training-based partial weight-supported treadmill walking training has been reported to aid in the recovery of walking abilities in stroke patients through muscle strengthening, lower limb stabilization, balance, and gait retraining [34]. When safety equipment is worn during treadmill walking training, abnormal gait patterns improve, and stride length and speed increase [35]. Furthermore, using an anti-gravity treadmill allows for weight-bearing exercises during the early post-surgery stages [36]. During early weight-bearing exercises, the patient’s weight can be adjusted up or down by 1%, allowing for pain control and exercise intensity regulation [37]. Another advantage of this training method is the presence of safety features, which help prevent falls or slips during exercise, ensuring safety throughout the training process [38]. For these reasons, robot-assisted walking training (RAGT) is currently gaining prominence. Using robots, it is possible to conduct training in various environments and positions. RAGT, in conjunction with treadmills, is being used for gait retraining and pattern training [39].

The key elements of PRT involve the provision of sufficient resistance and the application of a training program over a sufficient duration with a gradual increase in resistance and strength [40]. Recently, the frequency of use of RAGT devices in Parkinson’s rehabilitation protocols has been gradually increasing [41]. Since RAGT provides a cue of continuous and intensive sensory feedback by applying kinematic parameters, it has the advantage of being able to add training or adjust the difficulty level for individual patients. In addition, in the case of RAGT, safety has been confirmed and its feasibility for treatment has been positively evaluated, so the use of RAGT will gradually increase [42].

Treatment for advanced stages of PD differs significantly from treatment in the early stages [43]. It focuses on overcoming severe non-motor symptoms, including hallucinations, psychosis, dysphagia, urinary incontinence, orthostatic hypotension, and postural instability [44]. As a result, a multidisciplinary team approach is emphasized, addressing individualized care based on the patient’s condition [45]. While treatment guidelines are comprehensive and precise, the need for tailored therapies for each individual patient is still recommended [46]. This approach involves a careful evaluation of substantial resources, including specialized medical systems, personnel, interdisciplinary collaboration, and synchronized medical procedures [44,45,46,47]. Unfortunately, a shortage of healthcare personnel is an ongoing issue both domestically and globally, and robotic-based medical technology is emerging as one of the most promising solutions to this problem [48].

Various studies have been conducted on effective rehabilitation for Parkinson’s patients. However, research on symptom improvement and enhancing independent walking function remains limited. This study began with the hypothesis that physical therapy approaches could be effective in alleviating individual symptoms of PD and that the method of RAGT could improve walking abilities. It compares research from the past 20 years. Additionally, by comparing the advantages and disadvantages of commonly used medical and pharmacological approaches with physical therapy approaches, it is anticipated that treatment methods can be varied according to needs. Therefore, this research study could serve as a cornerstone for rehabilitative approaches aimed at enhancing muscle strength, balance, and walking abilities in Parkinson’s patients.

**Figure 1:**
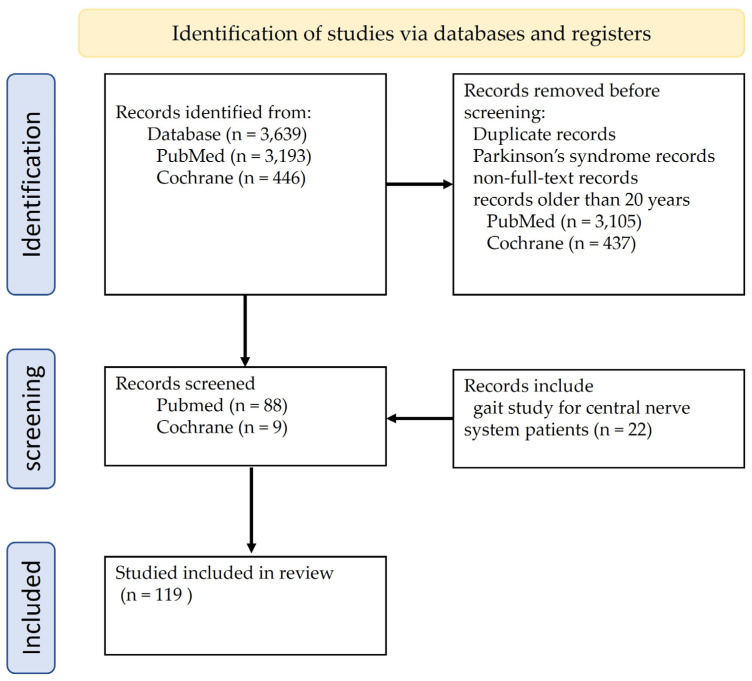
Flow diagram representing the study selection process.

This study analyzed common treatments and training methods for walking training applied in patients with Parkinson’s disease (PD) based on articles sourced from the PubMed and Cochrane databases. Initially, 3193 studies from PubMed and 446 from Cochrane were reviewed. Studies on Parkinson’s syndrome that were not full texts, over 20 years old, or had duplicate data were excluded. Additionally, 22 studies related to walking training applied to central nervous system patients were added, resulting in a total of 119 studies being used to compare treatment methods for PD. The above content is summarized and depicted in Figure 1.

## 2. Surgical Intervention and Pharmacotherapy

### 2.1. Surgical Intervention

There are various surgical treatment options for PD, primarily including deep brain stimulation (DBS) and implants. These procedures are typically considered for patients who do not respond well to medication therapy or experience severe side effects. Following surgical procedures for PD, several precautions should be observed. Even after surgery, symptoms may not be completely resolved, and there might be instances of transient exacerbation. Therefore, long-term monitoring is imperative, involving regular physician visits and appropriate testing. Additionally, medication therapy may require adjustment post surgery, and adherence to implant management guidelines is essential if implants such as deep brain stimulation (DBS) are utilized. Moreover, maintaining adequate physical activity and preventive healthcare measures post surgery is crucial. Adhering to these precautions will enable PD patients to maximize the potential benefits of surgery. However, continuous consultation with physicians to review symptoms and health status post-surgery remains imperative. In addition, placebo surgery, also known as sham surgery or simulated surgery, involves performing a surgical procedure that mimics the actual intervention being studied but without the therapeutic component. In the context of PD, placebo surgery is often used in clinical trials evaluating the efficacy of surgical interventions such as DBS [49].

#### 2.1.1. DBS

DBS involves implanting electrodes into specific areas of the brain to deliver electrical stimulation, which helps alleviate the symptoms of PD [50]. The electrodes are implanted into deep brain regions, and the intensity and frequency of the electrical stimulation can be adjusted using an external programmable device [51]. DBS can provide benefits such as improvements in motor function, reduction in motor fluctuations, and decreased medication usage. DBS offers significant improvement in motor symptoms, such as tremors, rigidity, and bradykinesia, providing enhanced quality of life for patients [52]. It allows for adjustable stimulation parameters, enabling personalized treatment tailored to individual patient needs. DBS can reduce the need for high doses of medication, thereby minimizing medication-related side effects. The effects of DBS are reversible, as the electrodes can be removed if necessary [53].

On the other hand, DBS surgery carries the risks associated with brain surgery, including infection, bleeding, and neurological deficits. There is a risk of hardware-related complications, such as electrode migration or malfunction, which may require additional surgical interventions [53,54,55]. Patients may experience adverse effects related to stimulation, such as speech or cognitive changes, although these are usually reversible and can be managed with adjustments to stimulation settings [56]. DBS is not a cure for PD and does not halt disease progression; it primarily provides symptom relief.

#### 2.1.2. Neurosurgical Procedures

These surgeries may include cortical stimulation, brain magnetic stimulation, brain surgery, cell transplantation, among others. However, these procedures are primarily considered for patients who have failed alternative therapies [57].

Certain neurosurgical procedures, such as cell transplantation, hold the potential for disease-modifying effects by replacing damaged neurons with healthy ones. These procedures may offer alternative treatment options for patients who do not respond to conventional therapies [58]. However, neurosurgical procedures are often invasive and carry risks inherent to brain surgery, including infection, bleeding, and neurological deficits. The efficacy and safety of some neurosurgical procedures, such as cell transplantation, are still under investigation, and long-term outcomes remain uncertain [59]. Not all patients are suitable candidates for neurosurgical interventions, and careful patient selection is necessary to minimize risks and optimize outcomes.

### 2.2. Pharmacotherapy

#### 2.2.1. Levodopa

Levodopa is converted into dopamine in the brain, increasing dopamine levels and thereby improving symptoms of PD, primarily manifested through improvements in motor function [60]. Prolonged use of levodopa may lead to motor fluctuations, dyskinesias, and motor instability. Additionally, levodopa can cause side effects such as dopamine dysregulation syndrome, hallucinations, and involuntary movements [61]. Levodopa is typically administered orally for symptom relief. It is commonly taken before meals or with food to maximize effectiveness. The appropriate dosage and dosing frequency of levodopa are determined through consultation with a healthcare professional to optimize its duration of action and minimize side effects [61,62].

#### 2.2.2. Dopamine Agonists

Dopamine agonists stimulate dopamine receptors in the brain, increasing dopamine levels similarly to levodopa but with fewer motor fluctuations [63]. Dopamine agonists can cause neurological and psychiatric side effects including depression, confusion, hallucinations, as well as sensory disturbances and cardiac abnormalities such as irregular heartbeats [64]. Dopamine agonists can be administered orally or through transdermal patches. Oral medications are usually taken after meals, while transdermal patches are preferred when patients have difficulty with oral intake. They may be used as initial therapy or in combination with levodopa [61,62,63,64].

#### 2.2.3. MAO-B

MAO-B inhibitors inhibit the breakdown of dopamine, increasing dopamine levels and alleviating symptoms of PD [65]. Common side effects include psychiatric symptoms such as depression, anxiety, and confusion. Serious side effects may include hypertension and acute coronary syndrome. MAO-B inhibitors are taken orally, typically after meals [66]. They are often used in combination with levodopa to enhance its effects and reduce motor fluctuations [61,66].

#### 2.2.4. COMT Inhibitors

COMT inhibitors enhance the effects of levodopa, increasing dopamine levels and prolonging its effects while reducing motor fluctuations [67]. Common side effects include gastrointestinal disturbances and hormonal changes, while rare side effects may include cardiac abnormalities [68]. COMT inhibitors are taken orally, usually after meals. They are used alongside levodopa to enhance its effects and mitigate levodopa-induced side effects [61,64,68].

#### 2.2.5. Anticholinergics

Anticholinergics reduce acetylcholine levels, alleviating symptoms such as tremors and muscle rigidity in PD [69]. Common side effects are primarily central nervous system-related, including confusion, memory impairment, visual disturbances, and dry mouth. Additionally, anticholinergics may exacerbate urinary problems in the elderly, necessitating caution during use. Anticholinergics are primarily administered orally [70]. They are generally taken after meals, and dosages may be adjusted as needed under the guidance of a healthcare provider [71].

#### 2.2.6. Appraisal of Pharmacotherapy in PD

Pharmacotherapy serves as a cornerstone in the management of PD, offering symptomatic relief and potentially slowing disease progression. Here, we discuss the advantages and disadvantages of commonly used medications [13,14,15,60,61,62,63,64,65,66,67,68,69,70,71].

Advantages: (1) Symptom relief: pharmacological agents such as levodopa, dopamine agonists, and MAO-B inhibitors provide significant symptomatic relief, improving motor function and quality of life for PD patients. (2) Disease modification: some medications, particularly MAO-B inhibitors and potentially levodopa, may exert neuroprotective effects, slowing the progression of PD and preserving dopaminergic neurons. (3) Customized treatment: pharmacotherapy allows for personalized treatment regimens tailored to individual patient needs. Healthcare providers can adjust medication dosages and combinations based on symptom severity and patient response, optimizing therapeutic outcomes. (4) Combination therapy: combining multiple medications, such as levodopa with dopamine agonists or COMT inhibitors, can enhance efficacy and mitigate adverse effects. This approach provides a multifaceted approach to symptom management [13,14,15,60,61,62,63,64,65,66,67,68,69,70,71].

Disadvantages: (1) Motor fluctuations: the long-term use of levodopa may lead to motor fluctuations, characterized by periods of enhanced motor function (on periods) alternating with periods of reduced function (off periods) or dyskinesias. (2) Non-motor side effects: pharmacotherapy for PD can be associated with various non-motor side effects, including psychiatric symptoms (e.g., hallucinations, psychosis), gastrointestinal disturbances, orthostatic hypotension, and cognitive impairment. (3) Tolerance and dyskinesias: prolonged use of dopaminergic medications, particularly levodopa, may result in the development of tolerance and dyskinesias, complicating treatment and necessitating dose adjustments. (4) Complexity of treatment: managing PD with pharmacotherapy requires careful monitoring and frequent adjustments to medication regimens. Patients may experience fluctuations in response, necessitating ongoing collaboration with healthcare providers [13,14,15,60,61,62,63,64,65,66,67,68,69,70,71].

### 2.3. Paletelet-Drived Growth Factor (PDGF)

As a type of neurotrophic factor (NTF), PDGF is an important mitogen and chemotactic agent. PDGF can be expressed in mesenchymal cells, osteoblasts, and vascular smooth muscle cells (VSMCs), playing active roles in neuronal development, differentiation, survival, and plasticity. Importantly, various NTFs have been shown to restore the dopaminergic nigrostriatal pathway, which is impaired in patients with Parkinson’s disease (PD). Research has demonstrated that platelets are a source of growth-promoting activity, and their extracts can promote the growth and proliferation of fibroblasts, smooth muscle cells, and glial cells. Therefore, PDGF is being studied as a potential therapeutic target in neurodegenerative diseases like PD, particularly in the areas of central nervous system neuroprotection, recovery promotion, and inflammation reduction [72,73,74,75].

## 3. Physical Therapy Approaches for Each Sign

### 3.1. Bradykinesia

Treatment of bradykinesia in PD through physical therapy plays a significant role in improving the patient’s strength, flexibility, balance, and walking abilities. Strength training helps improve muscle strength and function in Parkinson’s patients [3]. Various resistance exercises can be performed to strengthen both upper and lower body muscles. Examples include using resistance bands, hand weights, or other exercise equipment to perform a variety of exercises [28,29,30]. Stretching is crucial for Parkinson’s patients as their muscles tend to become stiff and tense. Regular stretching improves muscle flexibility and enhances joint range of motion, thereby alleviating movement slowness [76]. Since Parkinson’s patients often experience balance impairments, exercises to improve balance are essential [77]. These exercises may include standard squats, single-leg standing, or exercises involving shifting weight between the legs [78]. Gait training is crucial for managing bradykinesia in Parkinson’s patients. It helps improve patients’ walking patterns and alleviate movement slowness. Such training may involve various exercises and techniques aimed at modifying impaired walking patterns and enhancing walking abilities [79].

Physical therapy should be individualized and tailored to the specific symptoms and level of each patient. It should be conducted under the guidance of a professional and requires periodic assessment and adjustments. Long-term physical therapy plays a vital role in preserving function and enhancing the quality of life for Parkinson’s patients [28,29,30,76,77,78,79].

### 3.2. Muscle Rigidity

Treatment of rigidity in Parkinson’s patients through physical therapy focuses on improving muscle flexibility, reducing muscle stiffness, and promoting overall mobility [79].

Passive range of motion (PROM) exercises involve gently moving the patient’s joints through their full range of motion without the patient actively contracting their muscles [78]. These exercises help improve joint flexibility, reduce muscle stiffness, and alleviate rigidity. Active range of motion (AROM) exercises involve the patient actively moving their joints through their full range of motion. These exercises help improve muscle strength, flexibility, and coordination, thereby reducing rigidity [76,77,78].

Stretching exercises target specific muscle groups affected by rigidity. By stretching these muscles regularly, muscle stiffness can be reduced, and joint mobility can be improved. Stretching should be performed gently and held for an adequate duration to be effective [76]. The neurodevelopmental therapy (PNF and Bobath techniques) involves stretching and contracting muscles in specific patterns to improve flexibility, strength, and coordination. These techniques often involve a therapist applying resistance while the patient performs movements, promoting muscle relaxation and reducing rigidity [21,76,77,78,79,80,81].

Massage therapy can help alleviate muscle stiffness and reduce rigidity by promoting blood circulation, relaxing tense muscles, and improving tissue flexibility. Different massage techniques, such as deep tissue massage or myofascial release, may be used based on the patient’s needs [82]. Hydrotherapy or water-based therapy involves performing exercises in a pool or aquatic environment. The buoyancy of water reduces the effects of gravity, making movements easier and more comfortable for Parkinson’s patients with rigidity. Hydrotherapy can help improve muscle relaxation, flexibility, and overall mobility. Functional training involves performing activities that mimic daily tasks to improve functional mobility [81]. By practicing functional movements tailored to the patient’s needs, muscle coordination and flexibility can be enhanced, reducing rigidity during activities of daily living [79].

Physical therapy for rigidity in PD should be tailored to the individual’s specific symptoms, functional limitations, and goals. It is essential to work with a qualified physical therapist who can design a personalized treatment plan and provide guidance on proper exercise techniques and progression. Consistent and regular participation in physical therapy can help manage rigidity, improve mobility, and enhance overall quality of life for Parkinson’s patients [83].

### 3.3. Rigidity

Treatment for tremor in PD patients through physical therapy involves several methods. These treatments may vary depending on the patient’s symptoms and condition and are individually tailored following a comprehensive assessment. Below are explanations of commonly used tremor treatments in physical therapy [2,3,4]:

Relaxing the muscles and stretching them can help alleviate tremors. Physical therapists may target specific muscle groups through stretching and muscle relaxation techniques to relieve tremors [1,2,3]. Strength training exercises can strengthen the muscles, improve muscle control, and alleviate tremors [2,27,28,29,30,31,32]. A tailored exercise program targeting muscles affected by tremors can reduce their severity. Intensive physical therapy may be included in tremor treatment plans. This involves a physical therapist conducting an individual assessment of the patient’s tremors and developing customized exercise and treatment plans to alleviate tremors [84].

Functional education and using assistive devices are crucial for managing tremors. These devices can help alleviate tremors and improve functionality in daily life. Tremors can impair a patient’s balance and coordination [85]. Therefore, exercises aimed at improving balance and coordination are essential for managing tremors. If tremors restrict activity, using assistive devices for stability and protection can be beneficial. These devices minimize the impact of tremors and facilitate safe movement [2,3,4,84,85].

Treatment for tremors in PD patients through physical therapy should be tailored to each individual’s symptoms and condition. Collaborating with a physical therapist to develop and implement an individualized treatment plan is crucial for managing the patient’s tremors effectively [1,2,83,84].

### 3.4. Postural Instability

Treatment for postural instability in PD patients through physical therapy involves various approaches aimed at improving balance, stability, and posture [1,2,3,4]. Physical therapists design specific exercises to challenge and improve the patient’s balance. These exercises may include weight shifting, standing on one leg, tandem stances (standing with one foot in front of the other), and various balance board activities. Balance training helps enhance proprioception and strengthen core muscles, thereby improving postural stability [86]. Gait training focuses on improving the patient’s walking pattern and reducing the risk of falls. Physical therapists may incorporate techniques such as stride lengthening, step training, and obstacle negotiation to enhance gait stability and confidence. They may also provide cues and visual feedback to encourage proper gait mechanics [16,17,18,19,20,86].

Strengthening exercises target muscles involved in maintaining posture and balance, such as the core muscles, leg muscles, and muscles around the hips and pelvis [87]. Resistance training using the patient’s body weight, resistance bands, or free weights can help improve muscle strength and stability, contributing to better postural control. Coordination and agility drills aim to enhance the patient’s ability to react and adapt to postural challenges quickly. These drills often involve dynamic movements, such as side steps, crossover steps, and rapid changes in direction. By improving agility and coordination, patients can better respond to balance perturbations and prevent falls [18,19,20,87].

Physical therapists educate patients on the proper use of assistive devices such as canes, walkers, or orthoses to support their posture and stability during mobility. Training may include techniques for safely navigating obstacles, ascending and descending stairs, and getting up from a seated position. Assistive devices can provide additional support and confidence to patients with postural instability [2,18,19,20,30,88].

Overall, physical therapy plays a crucial role in managing postural instability in PD patients by addressing underlying impairments and promoting functional independence and safety in daily activities. Treatment plans should be tailored to the individual needs and abilities of each patient, with regular reassessment and adjustments as necessary to optimize outcomes [1,2,3,20,89].

### 3.5. Akinesia

Treatment of akinesia in Parkinson’s patients through physical therapy involves various interventions aimed at improving movement initiation, reducing bradykinesia, and enhancing overall motor function [79]. Physical therapists employ techniques to facilitate movement initiation in Parkinson’s patients. These may include cueing strategies, such as visual or auditory cues, to prompt the initiation of voluntary movements [2,79]. Additionally, therapists may use manual techniques or proprioceptive stimulation to facilitate the activation of motor pathways and encourage movement initiation [90]. AROM exercises are designed to improve joint flexibility and mobility, which can help alleviate akinesia symptoms [30,31,32]. These exercises involve the active movement of joints through their full range of motion, targeting specific muscle groups affected by akinesia. By promoting joint mobility, these exercises aim to enhance motor function and reduce stiffness [2,3,4,79].

Functional training involves practicing activities of daily living that require movement initiation, such as reaching, grasping, and walking. Physical therapists guide patients through these functional tasks, providing assistance and feedback to promote movement initiation and improve motor coordination [3,20,32,81]. By integrating movement initiation into functional activities, patients can translate therapy gains into real-life situations. Task-specific training focuses on practicing specific movements or tasks that are challenging due to akinesia. Therapists design tailored exercises that mimic real-life activities, such as reaching for objects or performing sequential movements. Through repetitive practice and task-specific feedback, patients learn to overcome akinesia-related movement difficulties and improve their ability to initiate motor actions [3,81,82,83].

Sensory stimulation techniques, such as tactile or proprioceptive stimulation, can help stimulate motor pathways and facilitate movement initiation in Parkinson’s patients [91]. Therapists may use sensory cues, such as textured surfaces or vibration, to enhance sensory input and promote motor response. By engaging sensory pathways, these techniques aim to bypass akinesia-related deficits and facilitate movement initiation [3,4,5,91].

Overall, physical therapy interventions for akinesia in Parkinson’s patients are designed to address movement initiation difficulties through a comprehensive approach that targets specific impairments and functional limitations. By incorporating various techniques and exercises tailored to individual needs, physical therapists aim to optimize motor function and improve overall quality of life for patients with PD [3,4,81,82,83,91].

## 4. Physical Therapy Approaches for Gait

One of the physiotherapy approaches to PD is electrical therapy, which involves stimulating the muscles with electrical impulses to improve movement. Therefore, electrical stimulation can be used to stimulate the muscles for walking and provide muscle re-education. This method promotes muscle activity, alleviating muscle stiffness and facilitating smoother movements [92]. There are various forms of electrical therapy, but the most commonly used ones for PD are electromyography (EMG) therapy and electromyographic biofeedback therapy. EMG therapy utilizes sensors to measure and record muscle activity. These sensors monitor and detect muscle activity, delivering appropriate electrical stimulation. This helps to promote muscle activation and reduce muscle stiffness. Electromyographic Biofeedback therapy monitors electromyographic activity and provides real-time feedback to improve muscle activity [93]. Patients can visualize or hear their electromyographic signals, increasing muscle usage and promoting more effective movements. Electrical therapy is typically used in conjunction with other treatment methods and is tailored to the individual patient’s condition and symptoms [94]. However, consultation with a professional is necessary before undergoing electrical therapy, and a personalized treatment plan should be established, considering the patient’s physical condition and other factors [2,3,92,93,94].

The application of PNF and Bobath techniques in gait training in PD patients may vary slightly from those applied to patients with hemiplegia [21,90]. However, these techniques can be beneficial for improving the gait of PD patients. PNF aims to strengthen muscles and increase the active range of motion, utilizing the muscles in a cross-pattern manner. When applied to PD patients, PNF can focus on improving gait patterns and enhancing balance. For example, utilizing muscles in a cross-pattern manner to strengthen leg muscles can promote flexibility and enhance movement for improving gait patterns in PD [21,80,90]. The Bobath technique is developed to treat functional impairments of the central nervous system, focusing on improving movement and muscle control. In the case of PD, the Bobath technique can assist in adjusting gait patterns and strengthening balance. It helps patients maintain proper gait patterns and improve muscle control to enhance stability. Additionally, the Bobath technique can strengthen proprioception and muscle control to adjust gait movements and enhance stability [21,81].

These techniques are often used in conjunction with other physical therapy methods to improve gait in PD patients and should be applied appropriately under the guidance of a professional. Moreover, treatment approaches should be adjusted based on each patient’s individual circumstances and symptoms [20,21,80,81].

To safely walk in the community, individuals typically need a walking ability of approximately 1.3 to 1.8 m/s, and a minimum of at least 0.8 m/s [95]. To increase the walking ability of Parkinson’s patients, several physical therapy methods are commonly used [83]. Strength training helps strengthen the muscles of Parkinson’s patients, improving their walking ability. Various strength exercises can strengthen the muscles of the legs and pelvis. Balance training is crucial for improving the balance of Parkinson’s patients [96]. Various balance exercises and movements are performed to improve balance, enhancing stability during walking. Gait training is important for improving walking patterns and efficiency. It includes various exercises and activities that teach walking techniques and improve walking movements [16,17,18,19,20,97]. Considering environmental factors during gait training is important. Creating an appropriate walking environment and removing obstacles help patients walk safely and comfortably [98]. It is important for Parkinson’s patients to use safety equipment, such as ankle foot orthoses, during gait training. Using assistive devices or protective gear helps protect the patient’s safety and supports walking [16,99].

PRT for RAGT in Parkinson’s patients involves gradually increasing resistance provided by the robotic device during walking [41]. This method aims to strengthen the muscles and improve walking abilities. During the exercise, the robotic device applies resistance to the legs while the patient walks, encouraging the patient to respond to the resistance [100]. It starts with minimal resistance and gradually increases it over time to enhance muscle strength. This approach helps strengthen the muscles and improve the stability and mobility required for walking, leading to a more natural walking pattern [24,26,41,100]. Additionally, PRT may promote neuroplasticity in the brain, which can further enhance patients’ walking abilities [101,102,103]. In conclusion, PRT for RAGT in Parkinson’s patients is an effective method for improving muscle strength and walking abilities. It can enhance the quality of life and promote independence in daily activities for patients [104,105].

RAGT is a specialized form of gait training that utilizes robotic technology to improve walking ability in PD patients [41,104]. During RAGT, robotic devices attached to the patient’s legs provide support and guidance during walking, helping to enhance muscle strength, improve balance, and increase walking capacity. These devices can offer various forms of assistance, such as stabilizing the patient’s legs and guiding their movement to facilitate smoother walking [106]. Additionally, RAGT can provide individualized therapy tailored to each patient’s needs, delivering different exercises and walking patterns to achieve optimal therapeutic effects [100,105,106]. Furthermore, the training allows for real-time monitoring and adjustment of the patient’s progress, leading to more effective outcomes. Overall, RAGT offers many benefits to PD patients with walking impairments, helping to enhance their walking abilities and improve their quality of life [41,100,107].

RAGT offers several advantages for Parkinson’s patients. Firstly, it provides precise and customizable assistance tailored to each patient’s needs, enabling targeted rehabilitation. Additionally, these devices can offer consistent and repetitive training sessions, essential for motor learning and functional recovery. Furthermore, they provide real-time feedback on gait parameters, allowing therapists to monitor progress accurately [106,107,108]. However, there are some limitations to consider. RAGT often requires specialized equipment and trained personnel, which may limit its accessibility and affordability. Moreover, some patients may find robotic assistance less engaging or motivating compared to conventional therapy. Lastly, while robotic devices offer assistance, they may not fully replicate the complex dynamics of natural walking, potentially limiting their effectiveness in real-world scenarios. Despite these drawbacks, RAGT remains a valuable tool in Parkinson’s rehabilitation, offering targeted and consistent therapy to improve walking abilities and enhance overall quality of life [100,108].

Various types of robotic devices are used in RAGT. These robotic devices provide stable support to the patient’s legs and guide movement during walking, aiding in improving walking abilities. Some commonly used robotic devices include the following: (1) the Lokomat, a robotic device attached to the lower body, simulating walking and guiding leg movements to enhance walking abilities [109]; (2) the G-EO System, which controls lower body movements to simulate walking and offers various walking patterns and training modes to provide personalized therapy tailored to the patient’s needs [108]; (3) ReWalk, a robotic exoskeleton device developed to support walking in lower limb paralysis patients which controls and guides leg movements to improve walking abilities [109]; and (4) Hybrid Assistive Limb (HAL), an exoskeleton robotic device that enhances muscle activity and guides walking to improve walking abilities [110,111]. These robotic devices each have their own advantages and characteristics and can be selected based on the patient’s condition and treatment goals. They serve as effective tools in RAGT, contributing to improving walking abilities and enhancing patients’ quality of life [41,100,104,105,106,107,108,109,110,111,112].

Anti-gravity walking training devices are a specialized piece of equipment used in gait rehabilitation, providing a weightless environment during walking sessions [113]. Representative examples of such devices include the AlterG [114]. This training simulates walking by reducing the body’s weight in mid-air. It is primarily conducted using specialized equipment or facilities that offer a reduced-gravity environment [115]. These devices typically support both individual and group training sessions, reducing physical load and increasing stability during walking practice. The aim of anti-gravity training is to improve gait patterns and strengthen muscles, contributing to enhanced walking ability by improving body posture and movement [36,113,114,115].

Aquatic walking training is a rehabilitation program that involves practicing walking in water, utilizing the unique environment provided by exercising in water [116]. This training is primarily conducted in swimming pools or aquatic therapy facilities, where patients perform underwater walking with the assistance of special aids or support from caregivers. This training reduces the burden on joints and muscles by utilizing the buoyancy of water to support the body [117]. Additionally, the resistance of water helps strengthen the muscles and improve muscle strength, balance, and range of motion [116,118]. Exercising in water reduces the risk of collisions and injuries while aiding in the improvement of walking and posture. Therefore, aquatic walking training is recognized as a beneficial rehabilitation program for musculoskeletal rehabilitation [116,117,118,119].

## 5. Conclusions

The treatment of PD requires a multidimensional approach, as the severity of symptoms varies among patients. Therefore, approaches to treatment may differ based on the severity of each patient’s symptoms. Tailoring treatments to address each symptom can play a crucial role in improving walking abilities. This study comprehensively examines the current medical, pharmacological, and physical therapy approaches applied to Parkinson’s patients. Current treatments for Parkinson’s patients aim to promote independent living. Accordingly, surgical interventions, pharmacological treatments, and physical therapy approaches are crucial for improving patients’ walking abilities and reducing dependency.

## Data Availability

Not applicable.
